# Predicting the Toxicity of Ionic Liquids toward Acetylcholinesterase Enzymes Using Novel QSAR Models

**DOI:** 10.3390/ijms20092186

**Published:** 2019-05-02

**Authors:** Peng Zhu, Xuejing Kang, Yongsheng Zhao, Ullah Latif, Hongzhong Zhang

**Affiliations:** 1 School of Materials Science and Energy Engineering, Foshan University, Foshan 528000, China; yszhao@sjtu.edu.cn; 2 School of Material and Chemical Engineering, Zhengzhou University of Light Industry, Zhengzhou 450001, China; xuejing_kang@hotmail.com; 3 Department of Chemical Engineering, University of California, Santa Barbara, CA 93106-5080, USA; 4 Department Beijing Key Laboratory of Ionic Liquids Clean Process, Key Laboratory of Green Process and Engineering, State Key Laboratory of Multiphase Complex Systems, Institute of Process Engineering, Chinese Academy of Sciences, Beijing 100190, China; latifucas@hotmail.com

**Keywords:** toxicity, ionic liquids, acetylcholinesterase enzyme, extreme learning machine, multiple linear regression

## Abstract

Limited information on the potential toxicity of ionic liquids (ILs) becomes the bottleneck that creates a barrier in their large-scale application. In this work, two quantitative structure-activity relationships (QSAR) models were used to evaluate the toxicity of ILs toward the acetylcholinesterase enzyme using multiple linear regression (MLR) and extreme learning machine (ELM) algorithms. The structures of 57 cations and 21 anions were optimized using quantum chemistry calculations. The electrostatic potential surface area (*S*_EP_) and the screening charge density distribution area (*S*_σ_) descriptors were calculated and used for prediction of IL toxicity. Performance and predictive aptitude between MLR and ELM models were analyzed. Highest squared correlation coefficient (*R*^2^), and also lowest average absolute relative deviation (AARD%) and root-mean-square error (RMSE) were observed for training set, test set, and total set for the ELM model. These findings validated the superior performance of ELM over the MLR toxicity prediction model.

## 1. Introduction

With ever-increasing demand for clean energy and stricter environmental regulations, scientists have always strived for sustainable and green chemical products [1]. The apparent benefits of ionic liquids (ILs) has spurred intensive research in order to replace the conventional hazardous solvents for broad scale industrial applications, such as organic reaction [2,3,4,5,6,7,8], catalytic processes [9,10,11,12], gas separation [13,14,15,16], storage materials [17,18,19,20], etc. Among the unique properties of ILs, negligible vapor pressure has primarily attracted the attention of many researchers, and the field has sought improvements in positively affecting overall efficiency of many chemical processes under the principles of green chemistry [21,22].

State-of-the-art research shows that the previously accepted advantage of low toxicity for ILs has proved to be overestimated, meaning that to some extent ILs in reality pose hazard potentials to humans and environment [23]. Rogers [22] et al. challenged the notion of green ILs because of their hazardous properties, e.g., unknown toxicity and stability. Meanwhile, Jastorff [24] et al. discussed the toxicity and ecotoxicity of ILs using quantitative structure-activity relationships (QSAR), which could be used to aid the rational design of optimal solvents from a technical and environmental perspective. Docherty [25] et al. found that ILs with hexyl- and octyl-imidazolium and pyridinium bromides had significant antimicrobial activity to pure cultures of Saccharomyces cerevisiae, Escherichia coli, Bacillus subtilis, and others. Later, Pang [26] et al. reviewed the environmentally relevant issues of ILs, such as environmental application and toxicity. Karunanithi [27] et al. studied the life cycle of aquatic ecotoxicity impacts for five common ILs by integrating physical properties, toxicity data, and transport parameters in the model. For the first time, they reported the freshwater ecotoxicity characterization factors for ILs. They found that an average of 83% of ecotoxicity impact was due to chemicals released during the upstream synthesis steps, while the remaining 17% ecotoxicity was related to the life-cycle energy consumption. All these findings underscore the need to develop sustainable and nontoxic ILs in future research. Unlike other basic physicochemical properties, such as density, viscosity, thermal stability, etc., to the best of our knowledge, research studies on the toxicity of ILs are still insufficient, and therefore need to be addressed further. In this regard, key developments in toxicity of ILs will highlight the designable feasibility of ILs to be environmentally benign with huge potential benefits for sustainable and green chemistry [28]. On the other hand, it is worthy to emphasize that around 10^8^ ILs are accessible due to the combination of enormously different cations and anions. Consequently, challenges have been encountered when investigating the toxicity of ILs [28,29]. To deal with these issues, it is highly necessary to develop efficient models to predict the toxicity of ILs with high accuracy.

Recently, QSAR studies have been reported in correlation with, and prediction of, properties of ILs [30,31,32,33,34,35,36]. QSAR is a powerful tool for accurately predicting the physical and chemical properties when applied to a set of molecular descriptors. In this regard, QSAR is supposed to be useful for estimating and screening chemical compounds for specific applications, having the desired characteristics for elucidating the underlying relation between micro-structures and macro-properties. Since the pioneering studies by Jastorff and co-authors [37], research attempts have been devoted to improving understanding and estimating the toxicity of ILs [30,38,39,40,41,42]. In subsequent years, Pereira et al. presented their toxicological assessment of a group of environmentally-friendly ILs with benign cholinium cations and linear alkanoate anions, using filamentous fungi as model eukaryotic organisms [39]. They found that the toxicity of ILs was increased with elongation in the linear chains of the anion, while branching resulted in reduced toxicity due to depressed lipophilicity [39]. Similar conclusions were drawn by Lima and Coutinho [43]. In order to further these studies, Ranke and co-workers studied up to 74 ionic liquids with different cations and anions to show the influence of cation lipophilicity on the cytotoxicity in *IPC-81* leukemia cells of rats. They found that substituents in the cation increased the toxicity of ILs [41]. Torrecilla et al. developed QSAR models based on MLR and neural network for prediction of toxicological effects of 96 ILs on *IPC-81* [42]. Romero and co-workers studied EC_50_ values of each compound in an aqueous solution using the microtox standard procedure. They found that the short length of the R_2_ side chain on imidazolium cation was favorable to diminish the toxic effect, while anions had a minor effect on the toxicity of ILs [44]. Yan et al. investigated the toxicity of ILs in acetyl cholinesterase enzyme (AChE) by the QSAR method using topological indices based on the atom characters. Their results demonstrated that the MLR model was capable of predicting the logEC_50_ (AChE) of ILs by using a 177 unit training set and a 44 unit testing set of topological indices generated from cations and anions [45]. Recently, Singh et al. investigated the chemical attributes of a wide variety of ILs towards their inhibitory potential of AChE using a supported vector machine (SVM) and a cascade correlation network (CCN) [46]. Their results showed that the proposed QSAR models had more statistical confidence, especially with respect to external validation, which has not been focused upon in other studies. This work addresses the success in predicting different toxicity classes and precise toxicity end-points of ILs using proposed QSAR. Like other researchers, we have previously focused on establishing the toxicity database of ILs, including over 4000 pieces of data and using QSAR models based on quantum chemistry descriptors and an artificial intelligence algorithm to study the toxicity toward the *IPC-81* [40]. The results revealed that the nonlinear model developed by the SVM algorithm was more reliable in the prediction of toxicity of ILs. Until now, very limited information has been available on the toxicity of ILs towards AChE. Our main objective in this work was to enhance the understanding of IL’s toxicity towards AChE by using novel extreme learning machine (ELM) algorithms with better accuracy in a bid to shed light on designing novel environmentally benign ILs for future applications.

## 2. Results and Discussion

### 2.1. Quantitative Prediction of Multiple Linear Regression (MLR) Model

**Figure 1:**
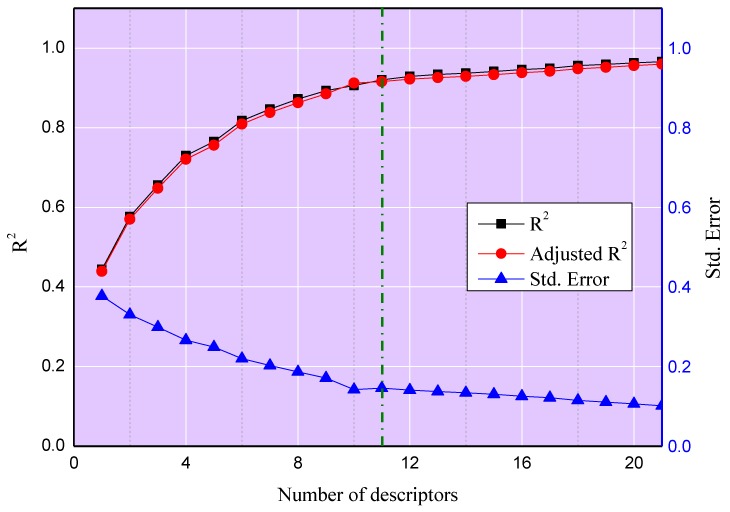
The squared correlation coefficient (*R*^2^) versus parameter number for the training data set.

To obtain the predictive model with an optimal number of descriptors, the stepwise regression approach was utilized to choose the effective input descriptors. As shown in Figure 1, the number of descriptors gradually grows as the squared correlation coefficient (*R*^2^) and adjusted squared correlation coefficient (*R*^2^_adjusted_) of the model grow, with simultaneous decrease in standard error (Std. Error). When the number of input parameters overtakes 11, the *R*^2^, *R*^2^_adjusted_, and standard error do not change evidently. Thus, the MLR model based on the optimal set of descriptors was determined. The final model is shown as Equation (1), where *P*_0_ stands for the intercept of the prediction model, while *C_i_* and *C_j_* mean the *i*th and *j*th coefficients respectively; *S_EP-__h_* is the electrostatic potential surface area for cations or anions at the electrostatic potential *h* while *S*_σ-_*_k_* means the screening charge density distribution area for cations or anions at the screening charge density *k*.

(1) $${\mathrm{logEC}}_{50}\left(\mathrm{AChE}\right)=P_{0}+\sum_{i=1}^{8}C_{i}S_{EP-h}+\sum_{j=9}^{11}C_{j}S_{\sigma -}{}_{k}$$

Details of the coefficients and the parameters, including the *t* values of the descriptors, can be found in Table 1. In total, eight *S_EP_* parameters and three *S_σ_* parameters were chosen for the model, where C is the cation and the numbers are the corresponding electrostatic potential and surface screening charge density, respectively. Since *t* values indicate the significance of the parameters, it can be seen that *S*_σ-C0.013_ is the most vital parameter for the prediction of the toxicity of ILs. The σ-profile range can be qualitatively divided into three main parts: the hydrogen-bond (HB) donor region (σ < −0.0082 e/Å^2^) and the HB acceptor region (σ > 0.0082 e/ Å^2^), as well as the non-polar region (−0.0082 e/Å^2^ ≤ σ ≤ 0.0082 e/Å^2^). Therefore, it can be concluded that the larger the *S*_σ-C0.013_ value of the ILs, the lower the toxicity of the ILs. The *S*_EP-C36.25_ and *S*_EP-C82.75_ are the second and third crucial parameters, while their coefficients are 0.361 and −0.213, respectively. It should be mentioned that all the selected parameters are from cations, which illustrates that the cations of ILs have a vital influence on the toxicity of ILs, while the anions have little effect. This finding is consistent with the result that the cation makes the dominant contribution to toxicity for most commercial ILs [45].

**Figure 2:**
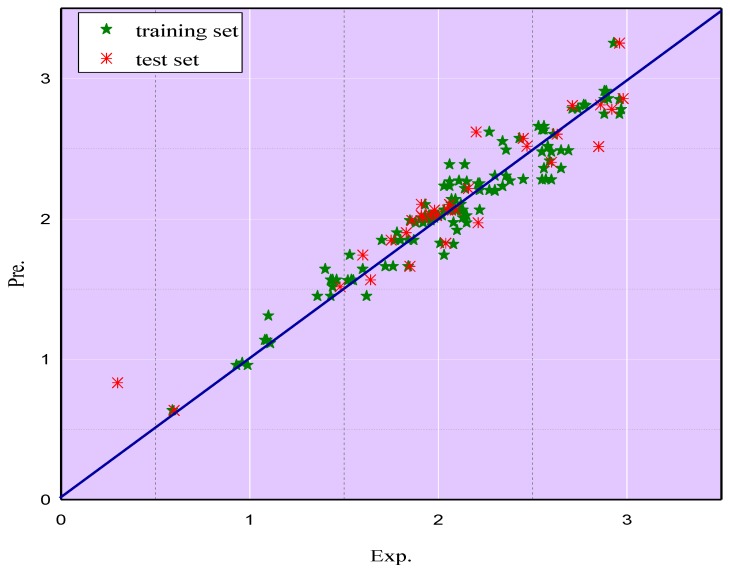
Comparisons of predicted the logEC_50_ values of ILs towards acetyl cholinesterase enzyme (AChE) (logEC_50_ (AChE)) by multiple linear regression (MLR) in external validation with the experimental logEC_50_ (AChE).

**Figure 3:**
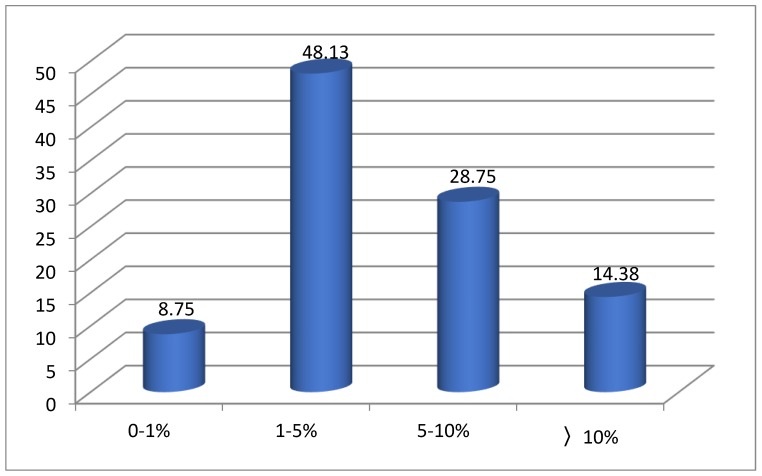
Percent of logEC_50_ (AChE) values in different absolute relative deviation (ARD%) ranges of the MLR model.

Experimental and predicted the logEC_50_ values of ILs towards acetyl cholinesterase enzyme (AChE) (logEC_50_ (AChE)) values of ILs (as shown in Supporting Information Table S1) via the MLR model for both the training set as well as the test set are plotted in Figure 2, where it can be seen that the predicted values are close to the experimental results. Figure 3 compares the absolute relative deviation (ARD%) within the range of logEC_50_ (AChE) values from the MLR model. It is evident that the largest proportion of the predicted values falls in the range of 1–5%, which accounts for 48.13%. Similarly, the percentages of predicted data fall in the range of 5–10% and over 10%, accounting for 28.75% and 14.38%, respectively. Only 8.75% of the predicted ARD% values are in the range of 0–1%. These conclusions showed the good performance of the MLR model to predict the toxicity of ILs.

### 2.2. Quantitative Prediction of Extreme Learning Machine (ELM) Model

**Figure 4:**
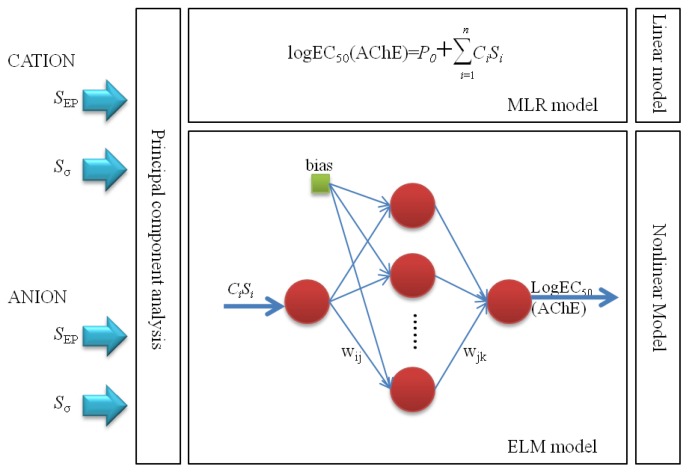
Scheme of linear and non-linear models programming methodology. The screening charge density distribution area (*S*_σ_) and the electrostatic potential surface area (*S*_EP_) of cations and anions were calculated employed to develop models optimal number of descriptors. The effective input descriptors were chosen by the stepwise regression approach and then the multiple linear regression (MLR) model was built with the obtained coefficients (*C_i_*) and selected descriptor (*S_i_*) to predict the the toxicity values (logEC_50_) of ionic liquids (ILs) towards acetyl cholinesterase enzyme (AChE). The extreme learning machine (ELM) comprising three parts—the input layer, hidden layer, and output layer—was established based on the same input descriptors. The parameters w_ij_ and bias from the input layer to hidden layer were generated randomly and the parameters w_jk_ were coefficients from the hidden layer to output layer.

**Figure 5:**
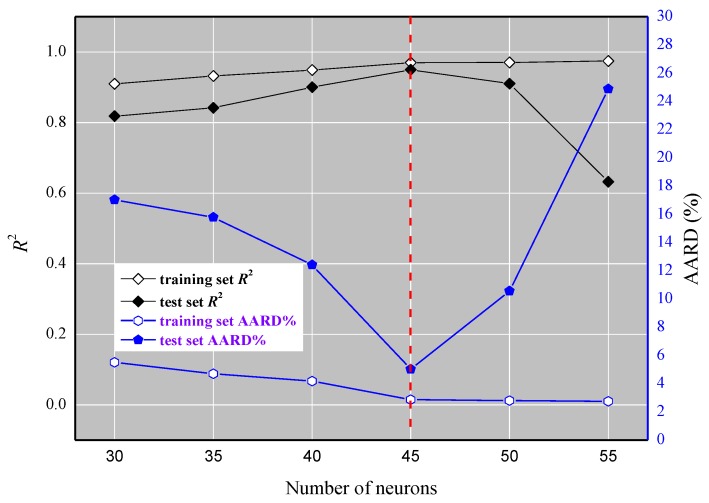
The *R*^2^ and average absolute relative deviation (AARD%) versus the parameter number for the ELM) model.

Based upon the foundation of 11 descriptors selected via stepwise regression method and the data sets from the former model, a more effective nonlinear ELM model was established. In the ELM model, there are three layers, as shown in Figure 4. The sine function in the ELM model acted as an activation function between the input and intermediate layers, while the linear function was from the intermediate neurons to the output variables. The bias and ω_ij_ produced randomly were the parameters between the first and intermediate layers. The ω_jk_ was the coefficient needed to calculate between the intermediate and final layer. After the determination of the functions, the next step was to acquire the ideal number of neurons between the input variables and intermediate neurons. As shown in Figure 5, in terms of the training set, the *R*^2^ value slightly increases with the growth in the number of neurons, whereas the average absolute relative deviation (AARD%) considerably drops when the number of neurons is less than 45, beyond which it dramatically rises. The variation tendency of *R*^2^ and AARD% for the test set are almost opposite to the growing number of neurons. Therefore, the best number of neurons (45) is acquired for the prediction the toxicity of ILs by the ELM model.

**Figure 6:**
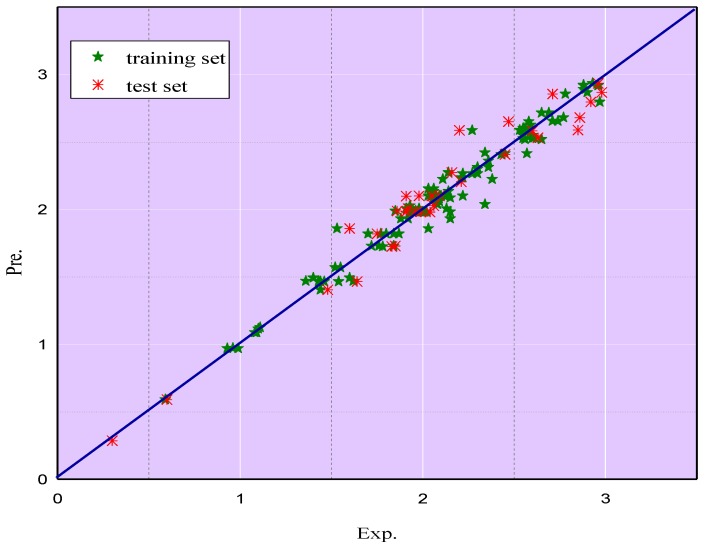
Comparisons of the predicted logEC_50_ (AChE) by ELM in external validation with the experimental logEC_50_ (AChE).

**Figure 7:**
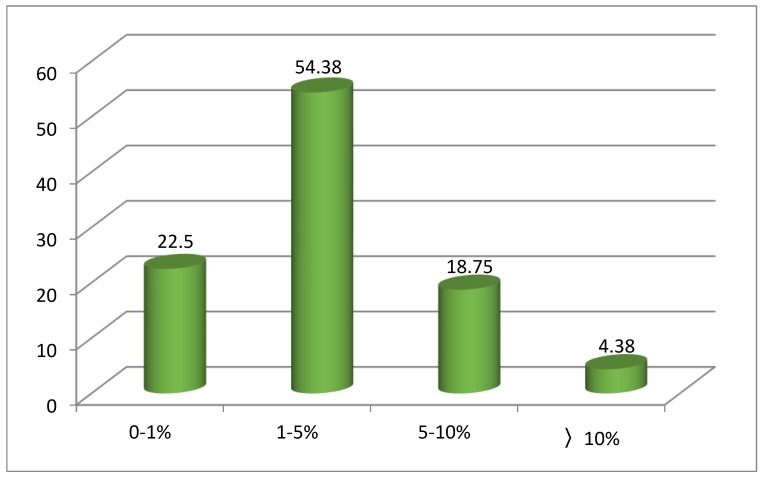
Percent of logEC_50_ (AChE) values in different ARD% ranges of the ELM model.

The comparisons of the predicted logEC_50_ (AChE) values by ELM (as shown in Supporting Information Table S1) in both the training and test sets with the experimental logEC_50_ (AChE) values are shown in the Figure 6, which reveals that the predicted values match well with the experimental data. Figure 7 illustrates the proportion of logEC_50_ (AChE) values in the different ARD% ranges of the ELM model. It is clear that the largest percentage of ARD% from the logEC_50_ (AChE) values is 54.38% within the range of 1–5%, followed by the 22.5% in the range of 0–1%. The percentage of ARD% of the logEC_50_ (AChE) values in the range of 5–10% and over 10% account for 18.75% and 4.38%, respectively. These results showcase the validity and reliability of the ELM model to predict the toxicity of ILs.

### 2.3. Comparison between Two Quantitative Structure-activity Relationships (QSAR) Models

As discussed above, satisfactory results are obtained for the two models having high *R*^2^ values. Detailed comparison of the statistical parameters of the training set, test set, and total data of different QSAR models are given in Table 2.

It is evident from Table 2 that the *R*^2^ values of the training set, test set, and total data using the ELM model are 0.969, 0.950, and 0.964, respectively, which are comparatively higher than of the *R*^2^ values of the MLR model, i.e., 0.920 for the training set, 0.914 for the test set, and 0.917 for the total set. This trend is valid for both AARD% and RMSE, meaning that the non-linear ELM shows better predictive performance than MLR. For example, AARD% values for the training set by the MLR and ELM models are 5.18 and 2.86, while the RMSE values are 1.534 and 0.950, respectively. A similar situation can easily be observed for the test set and all data sets using two models. Undoubtedly, the ELM algorithm is a powerful prediction tool with better accuracy.

In addition, according to the effective criteria used in the literature [47,48,49,50], a QSAR model with acceptable predictive ability should satisfy the conditions in Equations (2)–(5). As can be seen from Table 3, all the coefficients for both the MLR and ELM models satisfy the above-mentioned criteria, illustrating the good predictive power of the models in this study. To further verify the reliability of the models established in this study, we also predicted the toxicity values of five new ILs. The specific prediction results can be found in the Supporting Information (Table S2). It can be seen that the predicted values agree well with the experimental values, indicating that the models we built are reliable, and the ELM model has better prediction performance than the MLR model.

(2) $$R_{}^{2}>0.7$$

(3) $$\frac{R^{2}-R_{0}^{2}}{R^{2}}<0.1\;and\;0.85\;<k<1.15$$

(4) $$\frac{R^{2}-R_{0}^{\prime 2}}{R^{2}}<0.1\;and\;0.85\;<k\prime <1.15$$

(5) $$\left|R_{0}^{2}-R_{0}^{\prime 2}\right|<0.3$$

We compared our estimated results with the reported values, as summarized in Table 4. Our two QSAR models, MLR (*R*^2^ = 0.917) and ELM (*R*^2^ = 0.964), have higher *R*^2^ values compared with those of other models, except the Multiple linear regression (MLP) model (*R*^2^ = 0.973). Although MLP exhibits the highest value of *R*^2^, our models used less input parameters. Our models show AARD% values of 6.35 for MLR and 3.29 for ELM, which fall in the range of the reported values, i.e., 2.8 to 9.35 (Table 4). In addition, as can be seen from Table 4, the RMSE values of our models are also relatively low compared to the literature [45]. Taking these factors into consideration, our models are reliable and meaningful for accurate prediction of the toxicity of ILs towards AChE.

## 3. Framework

As we know, quantitative structure-activity relationships (QSAR) can be implemented in the form of linear and non-linear algorithms. The former are relatively easy and intuitively present the impact of each parameter upon the properties, while the latter are considered to be suitable for accurate prediction in real-world scenarios. In this work, the toxicity of ionic liquids (ILs) was predicted according to the scheme shown in Figure 4.

## 4. Dataset and Structural Descriptors

### 4.1. Dataset

Herein, toxicity data in acetyl cholinesterase enzyme (AChE) of 160 ILs was collected from the widely acknowledged ionic liquid (IL) database [40,51,52]. Concentration for 50% of maximal effect (EC_50_) values (μM) for AChE, written as log EC_50_, were converted into the form of a logarithm of half maximal effective concentration. The whole data set was divided into two parts: a training set of 80% ILs to build the model and a set of 20% ionic liquids (ILs) to evaluate the model’s predictability. Detailed information on the ILs studied in this paper, including the names, SMILES strings, log_10_(EC_50_) values, etc., can be found in Supporting Information.

### 4.2. Calculation of Descriptors

**Figure 8:**
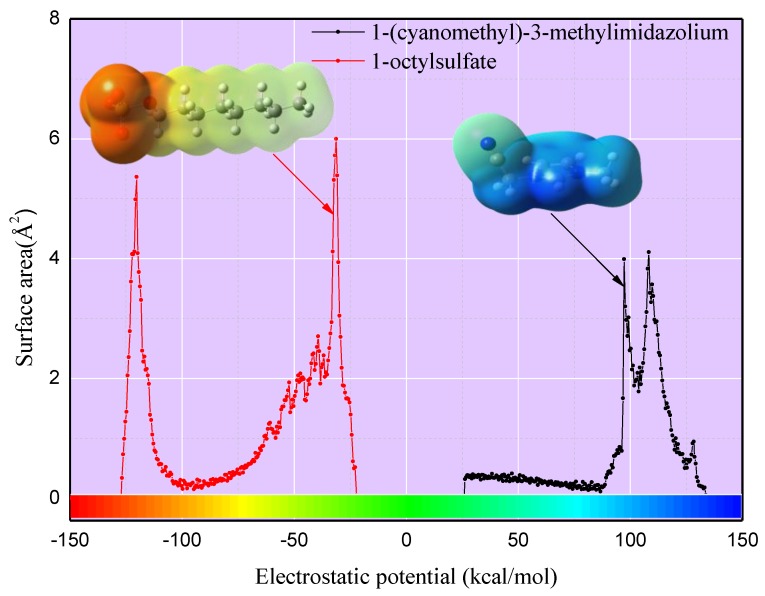
The electrostatic potential surface area (*S*_EP_) of representative cations and anions of ionic liquids (ILs).

**Figure 9:**
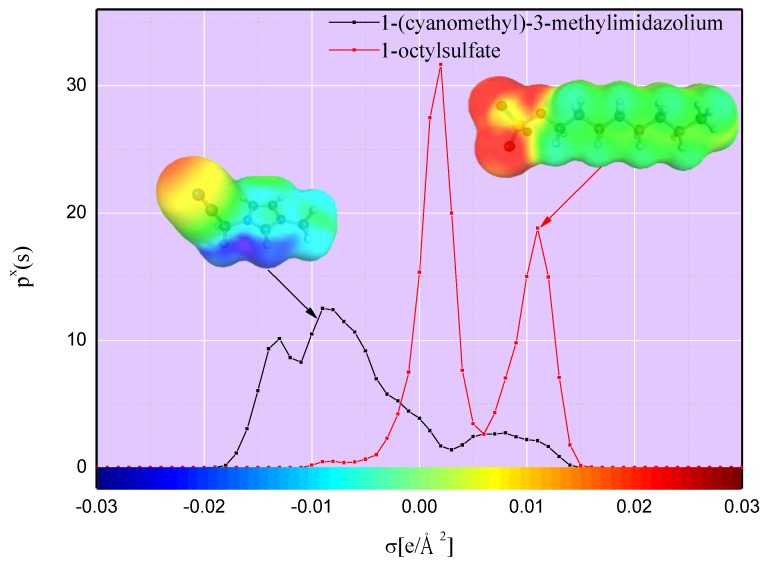
The screening charge density distribution area *(S*_σ_) of representative cations and anions of ILs.

As established, the related compounds are represented by theoretical molecular descriptors, which are of key significance for the predictive performance of the models [40]. The screening charge density distribution area (*S*_σ_), obtained from the histogram function of the σ-profiles given by the conductor-like screening model for real solvent (COSMO-RS) computation, is an a priori quantum chemistry descriptor that could quantitatively represent the molecule’s polar surface screen charge on the polarity scale [36]. The electrostatic potential surface area (*S*_EP_) of molecules refers to the surface areas of the molecules within the interval of the different electrostatic potential, and it can be used for the prediction of the material’s properties due to its rich information at the electron level. In this work, two descriptors *S*_EP_ and *S*_σ-profiles_ were employed to develop models and were calculated by different programs based on the optimal structures of cations and anions. First of all, the *S*_EP_ files of the corresponding cations were calculated using Multiwfn software with an electrostatic potential range of 0~150 kcal/mol, and was kept the same for the anions in the range of −150~0 kcal/mol, with a step size of 0.5 kcal/mol [53]. For example, the *S_EP-__C88.75_* means the molecular surface areas in the electrostatic potential scale of 88.5–89 kcal/mol. Then, the conductor-like screening model (COSMO) files of cations and anions were obtained using Gaussian 03 software [54]. Finally, *S*_σ-profiles_ files of corresponding cations and anions were calculated with a σ-profile ranging from −0.03 to 0.03 e/Å^2^ and with a step size of 0.001 e/Å^2^, respectively. We chose cation (1-(cyanomethyl)-3-methylimidazolium) and anion (1-octylsulfate) to present the representative S_EP_ and S_σ-profiles_, and the results were depicted in Figure 8; Figure 9, respectively. As shown in Figure 8, the darker shade of red for 1-(cyanomethyl)-3-methylimidazolium or blue for 1-octylsulfate indicates stronger polarity. Figure 9 presents a similar situation of electrostatic potential surfaces.

### 4.3. Multiple Linear Regression (MLR)

Multiple Linear Regression (MLR) is widely harnessed in quantitative structure-activity relationships (QSAR) to determinate the relationship between a dependent variable and various independent variables. The linear combination relationship of the independent variables is described by a set of coefficients in this model. The structural characteristics to the toxicity can be linked as follows:

(6) $${\mathrm{logEC}}_{50}=b_{0}+b_{1}x_{1}+b_{2}x_{2}+\cdot \cdot \cdot +b_{n}x_{n}$$

where *b*_0_ is the intercept and *b*_1_, *b*_2_, and *b_n_* are regression coefficients of the corresponding descriptors; *n* is the number of descriptors used in the equation in order to figure out the optimal regression model; *x* denotes the descriptor used to describe the chemical structure of the compound. MLR consists of stepwise selection of descriptors, which combines the forward and backward procedures.

### 4.4. Extreme Learning Machine (ELM)

Extreme learning machine (ELM) architecture was proposed for the first time by Huang et al., as a single hidden layer feed-forward neural network that has a very strong learning ability for application in basic classification and regression [55]. The most key characteristic of ELM is that it has faster learning speed compared with the traditional learning algorithms. In addition, ELM can be easily used and is a fast-learning and effective algorithm for the feed-forward neural network with single hidden layer interpolation capability and universal approximation capability, which are the two important characteristics of the feed-forward neural network. To some extent, ELM is similar to an artificial neural network (ANN), and its weights and biases in the first layer are randomly initialized and kept constant, while the weights of the second layer are selected by diminishing the least squared error. In practical applications, the training data set is mainly concerned with the specific issues [56]. The data sets include actual results and the related factors. Through iterations to finish the learning process, the impact factors and corresponding results were put into the ELM for training during the training process. Afterward, using the trained ELM, we only needed to input the training data set, similar to the influencing factors. It has been reported that ELM is feasible in many fields, such as protein sequence classification, regression problems, etc., to easily achieve good performance with fast speed [57]. Evidently, increasingly more research in this regard shows that excellent predictive performance can be achieved using the non-linear model developed by the ELM algorithm. More information on the theory and application of ELM can be found elsewhere [55,56,57].

### 4.5. Evaluation of Quantitative Structure-activity Relationships (QSAR)

In this work, several parameters were defined to evaluate the model performance, as measured by different metrics: adjusted squared correlation coefficient (*R*^2^_adjusted_), squared correlation coefficient (*R*^2^), average absolute relative deviation (AARD%), root-mean-square error (RMSE), absolute relative deviation (ARD%), the coefficients of calculated values versus experimental values ($R_{0}^{2}$), experimental values versus calculated values ($R_{0}^{\prime 2}$), slope *k* of the calculated versus experimental values, and slope *k’* of the experimental versus calculated values. The corresponding definition could be expressed in Equations (7)–(15).

(7) $$R^{2}=\frac{\sum_{i=1}^{N_{p}}(y_{i}^{\exp}-{\bar{y}}_{m}{)}^{2}-\sum_{i=1}^{N_{p}}(y_{i}^{\mathrm{cal}}-y_{i}^{\exp}{)}^{2}}{\sum_{i=1}^{N_{P}}(y_{i}^{\exp}-{\bar{y}}_{m}{)}^{2}}$$

(8) $$R^{2}{}_{\mathrm{adjusted}}=1-\frac{\left(1-R^{2})(N_{P}-1\right)}{N_{P}-p-1}$$

(9) $$\mathrm{AARD}(\%)=100\times \sum_{i=1}^{N_{P}}\left(\left|\frac{y_{i}^{\mathrm{cal}}-y_{i}^{\exp}}{y_{i}^{\exp}}\right|\right)/N_{p}$$

(10) $$\mathrm{RMSE}=\sqrt{{\sum_{i=1}^{N_{P}}\left(y_{i}^{{}_{\mathrm{cal}}}-y_{i}^{{}_{\exp}}\right)}^{2}/N_{P}}$$

(11) $$\mathrm{ARD}(\%)=\left|100\times (y_{i}^{{}_{\mathrm{cal}}}/y_{i}^{{}_{\exp}}-1.0)\right|$$

(12) $$R_{0}^{2}=1-\frac{\sum_{i=1}^{N_{p}}(y_{i}^{\mathrm{cal}}-ky_{i}^{\mathrm{cal}}{)}^{2}}{\sum_{i=1}^{N_{P}}(y_{i}^{\mathrm{cal}}-\bar{y^{\mathrm{cal}}}{)}^{2}}$$

(13) $$R_{0}^{\prime 2}=1-\frac{\sum_{i=1}^{N_{p}}(y_{i}^{\exp}-ky_{i}^{\exp}{)}^{2}}{\sum_{i=1}^{N_{P}}(y_{i}^{\exp}-k^{\prime }\bar{y^{\exp}}{)}^{2}}$$

(14) $$k=\frac{\sum_{i=1}^{N_{p}}y_{i}^{\exp}y_{i}^{\mathrm{cal}}}{\sum_{i=1}^{N_{P}}(y_{i}^{\mathrm{cal}}{)}^{2}}$$

(15) $$k^{\prime }=\frac{\sum_{i=1}^{N_{p}}y_{i}^{\exp}y_{i}^{\mathrm{cal}}}{\sum_{i=1}^{N_{P}}(y_{i}^{\exp}{)}^{2}}$$

where *y* means logEC_50_ (AChE) value of ILs while the “^cal^” and “^exp^” superscripts stand for calculated and experimental values, respectively. *N_p_* denotes the number of data points for the corresponding dataset; *ȳ_m_* represents the average log EC_50_ (AChE) value of ILs for all of the selected data.

## 5. Conclusions

Safe operation and benign utilization are crucial for ILs, therefore the scope of accurately predicting the toxicity of ILs is of vital interest for scientific and technological advancement. In this work, detailed data analyses of the toxicity of ILs were made based on the established database and novel QSAR models were developed for toxicity prediction of 160 ILs in AChE. Good correlation between the estimated and original data was observed for the training set, test set, and the total set. It was found that the newly used ELM model gave the highest *R*^2^ values and the lowest values for AARD% and RMSE. The results validated the superior performance of ELM for estimation of toxicity of ILs. This study will be helpful in design and screening of environmentally friendly commercial ILs.

## Figures and Tables

**Table 1:** Coefficients (*C_i_*), parameters (*S*_EP-*h*_ and *S*_σ-*k*_) and the *t* values for Equation (1).

***i***	**Coefficients-C*_i_***	**^1^ S_EP-*h*_**	**^3^*t***
1	−0.185	S_EP-C88.75_	−6.582
2	−0.266	S_EP-C35.25_	−6.731
3	0.361	S_EP-C36.25_	10.565
4	−0.213	S_EP-C82.75_	−10.501
5	−0.289	S_EP-C31.25_	−7.917
6	0.133	S_EP-C89.75_	4.936
7	0.204	S_EP-C64.25_	7.241
8	−0.142	S_EP-C53.25_	−5.498
***j***	**Coefficients-C*_j_***	**^2^ S_σ-*k*_**	***t***
9	0.537	S_σ-C0.013_	18.957
10	−0.102	S_σ-C-0.012_	−9.583
11	0.234	S_σ-C-0.016_	8.303
P_0_	2.712		

^1^ The electrostatic potential surface area (*S*_EP_) at the electrostatic potential *h*; ^2^ The screening charge density distribution area (*S*_σ_) at the screening charge density *k*; ^3^ *t* value stands for the importance of the parameter.

**Table 2:** Comparison of the statistical parameters by different quantitative structure-activity relationships (QSAR) models.

Model	Dataset	No.	^1^ *R* ^2^	^2^ AARD%	^3^ RMSE
MLR	Training	128	0.920	5.18	0.136
	Test	32	0.914	11.05	0.180
	Total	160	0.917	6.35	0.145
ELM	Training	128	0.969	2.86	0.084
	Test	32	0.950	5.02	0.134
	Total	160	0.964	3.29	0.096

^1^ Squared correlation coefficient (*R*^2^); ^2^ Average absolute relative deviation (AARD%); ^3^ Root-mean-square error (RMSE).

**Table 3:** The results of external validation of the test set in the multiple linear regression (MLR) and extreme learning machine (ELM) models.

Model	*R* ^2^	^1^ *k*	^2^ *k′*	^3^ *R*_0_^2^	^4^ $R_{0}^{\prime 2}$	*(R* ^2^ *-R*_0_^2^ *)/R* ^2^
MLR	0.914	0.9876	1.0058	0.9975	0.9996	−0.094
ELM	0.950	0.9944	1.0018	0.9996	1.0000	−0.051

^1^*k* is slope of the calculated versus experimental values; ^2^ *k′* is slope of the experimental versus calculated values; ^3^ the coefficients of calculated values versus experimental values ($R_{0}^{2}$); ^4^ the coefficients of experimental values versus calculated values ($R_{0}^{\prime 2}$).

**Table 4:** Comparisons of our work with the reported works.

Method	No. Parameter	*R* ^2^	AARD%	RMSE	Ref.
^1^ MLR	12	0.814	7.7	/	[42]
^2^ MLP	12	0.973	2.8	/	[42]
^3^ RB	12	0.842	7.1	/	[42]
MLR	17	0.877	9.35	0.212	[45]
MLR	11	0.917	6.35	0.145	This work
^4^ ELM	11	0.964	3.29	0.096	This work

^1^ Multiple linear regression (MLR); ^2^ Multilayer perceptron (MLP); ^3^ Radial-basis function (RB); ^4^ Extreme learning machine (ELM); / indicates that the RMSE value cannot be obtained from literature.

## Supplementary Materials

Supplementary materials can be found at https://www.mdpi.com/1422-0067/20/9/2186/s1.
